# Changes in the Cholinergic Innervation Pattern of Porcine Ovaries with Cysts Induced by Dexamethasone Administration

**DOI:** 10.1007/s12031-014-0239-1

**Published:** 2014-02-13

**Authors:** A. Kozłowska, M. Majewski, B. Jana

**Affiliations:** 1 Division of Reproductive Biology, Institute of Animal Reproduction and Food Research of the Polish Academy of Sciences, Tuwima 10, 10-748 Olsztyn, Poland; 2 Department of Human Physiology, Faculty of Medical Sciences, University of Warmia and Mazury, Warszawska 30, 10-561 Olsztyn, Poland

**Keywords:** Cholinergic innervation, Ovarian cysts, Gilts

## Abstract

We revealed earlier that induction of ovarian cysts in gilts by dexamethasone phosphate disodium salt (DXM) administration from the follicular phase of the estrous cycle (EC) changed the cholinergic innervation of the gonad. In the present study, the innervation of porcine ovaries by vesicular acetylcholine transporter (VAChT)-, neuronal nitric oxide synthase (nNOS)-, vasoactive intestinal peptide (VIP)- and somatostatin (SOM)-immunoreactive (IR) fibres, after induction of cystic changes from the middle luteal phase of the EC, was determined. The cystic changes were induced by DXM injections from days 7 to 21 of the EC, and 11 days later, the ovaries were collected. In the cystic ovaries, VAChT-, nNOS- and SOM-IR fibres were found around cysts and small tertiary follicles; nNOS-IR and also VAChT-IR fibres were observed near secondary follicles and veins; and VAChT- and nNOS-IR fibres were not found around cortical arteries. The number of VIP-IR fibres increased near the cysts and within the ground plexus, while the number of VAChT-IR fibres decreased within the medullar part of this structure. Thus, our study showed changes in the cholinergic innervation pattern of the porcine cystic ovaries induced from the middle phase of the cycle and confirmed that cystic ovary innervation depends partly on the phase of the EC in which the induction of cysts was started.

## Introduction

The porcine ovary receives its nerve supply from sympathetic, parasympathetic and sensory components of the peripheral nervous system (PNS). On the area of the ovary, parasympathetic nerve fibres that originate from the cranial part of the paracervical ganglion supply follicles (mainly primary and antral), corpora lutea (CL), blood vessels and interstitial gland and occur within the ground plexus. Besides acetylcholine (ACh), these fibres synthesize and release also nitric oxide (NO), vasoactive intestinal peptide (VIP) and somatostatin (SOM; Majewski 1997). The involvement of ACh and the aforementioned substances in the regulation of ovarian function has been demonstrated (Trzeciak et al. 1986; Dynarowicz and Dzięgielewski 1987; Masuda et al. 2001; Barszczewska and Jaroszewski 2004; Nestorović et al. 2008; Delgado et al. 2010).

Cystic ovarian disease (COD) is characterized by ovulatory failure and the presence of cysts, which causes abnormal estrous behaviour and infertility in farm animals. It has been reported that the prevalence of COD in cow herds varies from 5 to 30 % (Vanholder et al. 2006), while in sows from 2.4 to 40 % (see review of Cech and Dolezel 2007). Many possibilities are involved in COD development and maintenance. This disease results mainly from a dysfunction of the hypothalamic-pituitary-ovarian axis function (see review of Ahmed et al. 2003). However, more often, the participation of the PNS in the pathogenesis of ovarian cystic changes is documented. It was revealed that in rats, the steroid-induced augmentation in the sympathetic nerve activity and stress are able to cause a cystic condition similar to polycystic ovary syndrome (PCOS) in humans (Dorfman et al. 2003). The importance of the sympathetic nerve activity to the development and/or maintenance of ovarian cysts was indicated also in cows (Paredes et al. 2011). Similarly, in the studies performed on gilts, we have found that in the dexamethasone phosphate disodium salt (DXM)-induced cystic ovaries from the follicular (Jana et al. 2005) and middle luteal phase (Kozłowska et al. 2013) of the estrous cycle, the population of the sympathetic nerves is increased, accompanied by an increase in noradrenaline (NA) accumulation. Observed changes in the density of these nerves as well as in the content of NA were different depending on the phase of the estrous cycle in which the induction of the cysts was started. Moreover, our previous reports also show that both cholinergic (Kozłowska et al. 2008) and sensory (Kozłowska et al. 2011) innervation patterns were markedly changed in the porcine ovaries with DXM-induced cysts from day 16 or 7 of the estrous cycle, respectively, which can affect pathological gonad function.

It is well known that the morphological and functional changes of cystic ovaries are tightly bound with the kind of hormones inducing this pathological state, and they are also closely related to the phase of the estrous cycle in which administration of a hormone was started (Peter and Liptrap 1985; Frautschy and Liptrap 1988). Furthermore, under physiological conditions, ACh (Łakomy et al. 1984) as well as NO (Masuda et al. 2001) and VIP (Bruno et al. 2011) regulate the ovarian steroidogenesis depending on the stages of the estrous cycle in pigs and rats.

The above-mentioned data suggested that the cholinergic innervation pattern of cystic ovaries induced by DXM treatment from the middle luteal phase of the estrous cycle may be different from that observed after creation of cystic changes from the follicular phase of the estrous cycle (Kozłowska et al. 2008). Therefore, alterations in the distribution and density of nerve fibres containing vesicular ACh transporter (VAChT, as marker of ACh) and/or neuronal NO synthase (nNOS), VIP, and SOM were subjected to examination in the polycystic ovaries of gilts, induced by DXM treatment from the middle luteal phase of the estrous cycle.

## Materials and Methods

### Animals and Experimental Procedure

We followed the principles of animal care (NIH Publication no. 86-23, revised in 1985) as well as the specific national law on animal protection. The experimental procedures were approved by the Local Ethics Committee, University of Warmia and Mazury in Olsztyn (Agreement no. 36/N). The experiment was carried out on 12 crossbred gilts (Large White × Landrace), aged 7–8 months and of 90–100 kg body weight, with two controlled subsequent estrous cycles. Estrous cycles were monitored by using a boar tester. The animals were then individually housed in stalls, under conditions of natural light and room temperature (RT). They were fed with a commercial grain mixture and tap water ad libitum. The gilts were randomly assigned to one of the two groups: control, receiving saline (CON, n = 6), and DXM-treated (DXM, n = 6).

In the DXM group, the polycystic status of ovaries was induced according to the protocol described earlier by Gee et al. (1991) with the following modifications: before the ovulatory follicle selection, every 12 h from day 7 to day 21 of the first studied estrous cycle (i.e. throughout 15 consecutive days), the gilts received DXM (3.3 μg/kg of body weight, in total volume of 6 ml; Dexasone®, Norbrook Laboratory, Newry, UK). During the same period of time, animals of the CON group were injected with 6 ml of saline. The gilts were then slaughtered by electrical shock (ENZ 300 Metalowiec, Bydgoszcz, Poland) and exsanguinated on the expected 11^th^ day of the second studied cycle (i.e. 26^th^ day of the experiment). The ovaries were immediately dissected out, their weight and volume measured and the numbers of follicular structures, corpora lutea (CLs) and cysts estimated. The follicles were macroscopically divided into two sub-classes: small (1–3 mm in diameter) and medium (4–6 mm in diameter). Follicular structures exceeding 1.0 cm in diameter were classified as cysts (Nalbandov 1952). Morphological examination of the ovaries was particularly focused on the results described previously by Kozłowska et al. (2009) on the number of partly luteinized follicular cysts, a decrease in the number of small follicles and the lack of medium follicles and CLs. Afterwards, blocks of ovarian tissue were processed for further immunochemical studies as follows: they were fixed by immersion in Zamboni’s fixative for 30 min, washed in 0.1 M phosphate buffer (once every 3 days), stored in 18 % phosphate-buffered sucrose for 14 days and then frozen (−80 °C) and stored until sectioning.

### Immunohistochemistry

Cryosections from ovaries (10 μm, Reichert-Jung, Nuβloch, Germany) were subjected to double immunofluorescence staining technique. Sections were air-dried at RT for 45 min and rinsed (3 × 15 min) with phosphate-buffered saline (PBS, pH 7.4). Samples were then incubated with a blocking mixture containing 1 % Triton X-100 (Sigma-Aldrich, USA), 0.1 % bovine serum albumin (Sigma-Aldrich, USA), 0.05 % thimerosal (Sigma-Aldrich, USA), 0.01 % NaN_3_ and 10 % of normal goat serum (MP Biomedicals, USA) in 0.01 M phosphate-buffered saline for 1 h at RT. After washing (3 × 15 min), the sections were incubated with a mixture of primary antibody VAChT (rabbit, cat. no. H-V006, Phoenix Pharmaceuticals, working dilution 1:10,000), nNOS (mouse, cat. no. N2280, Sigma-Aldrich, USA, working dilution 1:1,000), VIP (mouse, cat. no. 9535-0504, Biogenesis, working dilution 1:2,000) and SOM (rat, cat. no. MAB354, Merck, working dilution 1:60) overnight in a humid chamber at RT. Following subsequent rinsing in PBS (3 × 15 min), the sections were incubated (1 h) with biotinylated donkey anti-rabbit IgG (cat. no. AP132B, Chemicon, UK, working dilution 1:1,000). A mixture of fluorescein isothiocyanate (FITC)-conjugated donkey anti-rat IgG or anti-mouse IgG-specific (1 h; cat. no. 712095153 and 715095150, respectively; both from Jackson ImmunoResearch Lab, USA, working dilution 1:400) and CY3-conjugated donkey anti-rabbit IgG-specific antisera (1 h; cat. no. 016160084, Jackson ImmunoResearch Lab, USA, working dilution 1:7,500) was added, and the sections were washed (3 × 15 min) and then coverslipped with carbonate-buffered glycerol (pH 8.6). The specificity of the primary antisera was tested as follows: sections were incubated with antibody that had been preabsorbed with synthetic antigen (10 μg of antigen per ml diluted antiserum); the primary antibody was omitted from the incubation; or normal rabbit, rat or mouse serum was substituted for the primary antibody.

Fluorescent nerve fibres were documented by conventional fluorescence microscopy (Olympus BX51 microscope equipped with epi-fluorescence and appropriate filter sets). The localization and density of intraovarian VAChT and/or nNOS, VIP and SOM nerve terminals were estimated around follicles, cysts, CLs, blood vessels, interstitial glands and within the autonomic ground plexus. In order to evaluate differences in the distribution pattern of perifollicular nerve fibres, ovarian follicles were microscopically classified according to Wulff et al. (2002) and Barboni et al. (2004) into the following classes: primordial—without granulosa cells; primary—surrounded by a single layer of cuboidal granulosa cells; secondary—with two or more granulosa cell layers without antral cavity; and tertiary—with antrum. Moreover, the tertiary follicles were divided into two size classes: up to 3 and 4–6 mm in diameter. As mentioned earlier, follicular structures exceeding 1.0 cm in diameter were classified as cysts (Nalbandov 1952). The mean number of VAChT-, nNOS-, VIP- and SOM-IR nerve fibres supplying the particular ovarian structures was counted on 54 randomly chosen ovarian sections from each group (9 ovarian sections multiplied by 6 animals). The fibres were counted using Merz grid within a selected area of the picture (around or within the studied structures; Pidsudko et al. 2011). Next, the mean number of VAChT-, nNOS-, VIP- and SOM-IR nerve fibres from all 54 ovarian sections from each group was calculated.

### Statistical Analysis

Statistical analysis was performed using one-way analysis of variance (ANOVA) followed by the Bonferroni test. All data points are presented as mean values ± SEM using In Stat GraphPad (San Diego, CA) software.

## Results

### Distribution and Density of VAChT-, nNOS-, VIP- and SOM-IR Nerve Fibres in the Control and Cystic Ovaries

Data concerning the localization and density of nerve fibres containing VAChT, nNOS, VIP and SOM are presented in Table 1.

**Table 1:** Mean (±SEM) numbers of VAChT-, nNOS-, VIP- and SOM-IR nerve fibres in the porcine ovaries of the control (CON) and DXM-treated (DXM) groups

Ovarian tissue	Studied substances							
	VAChT		nNOS		VIP		SOM	
	Group							
	CON	DXM	CON	DXM	CON	DXM	CON	DXM
Cortex								
Ground plexus	2.6 ± 0.7	3.0 ± 0.6	3.1 ± 0.4	3.5 ± 0.4	4.1 ± 0.3	15.3 ± 1.4 b.f.^***^	–	3.3 ± 0.3
Follicles								
Primordial	–	–	–	–	0.6 ± 0.2	–	–	–
Primary	–	–	–	–	–	–	–	–
Secondary	–	0.5 ± 0.2	–	1.0 ± 0.0	1.0 ± 0.0	–	–	–
Tertiary in diameter (mm)								
– to 3	–	3.8 ± 0.4	–	3.8 ± 0.4	1.0 ± 0.0	1.0 ± 0.0	–	1.0 ± 0.0
– 4–6	–	l.s.	–	l.s.	1.0 ± 0.0	l.s.	–	l.s.
Cysts	l.s.	18.1 ± 0.7	l.s.	18.1 ± 0.7	l.s.	3.0 ± 0.2^*^	l.s.	1.0 ± 0.0
Corpora lutea	0.8 ± 0.6	l.s.	–	l.s.	–	l.s.	–	l.s.
Arteries	0.8 ± 0.6	–	0.6 ± 0.2	–	3.0 ± 0.3	3.5 ± 0.4	–	–
Veins	0.6 ± 0.2	0.5 ± 0.2	–	1.0 ± 0.0	2.8 ± 0.3	–	–	–
Interstitial gland	1.0 ± 0.0	1.0 ± 0.0	1.0 ± 0.0	1.0 ± 0.0	1.0 ± 0.0	1.0 ± 0.0	1.0 ± 0.0	1.0 ± 0.0
Medulla								
Ground plexus	3.0 ± 0.6	0.5 ± 0.2^**^	3.3 ± 0.3	4.1 ± 0.3 b.f.	3.0 ± 0.3	16.8 ± 0.7 b.f.^***^	3.0 ± 0.6	15.7 ± 0.6^***^
Arteries	–	–	3.0 ± 0.3	–	3.0 ± 0.6	3.8 ± 0.3	–	–
Veins	–	–	–	–	2.6 ± 0.3	3.0 ± 0.3	–	–

*l.s*. lack of structures, *b.f*. bunches of fibres, – lack of fibres

*P < 0.05, **P < 0.01, ***P < 0.001 indicate significant differences between the examined groups for the same structures and between follicles measuring 4–6 mm in diameter in the CON and cysts in the DXM group

#### Cortex

**Figs. 1–7:**
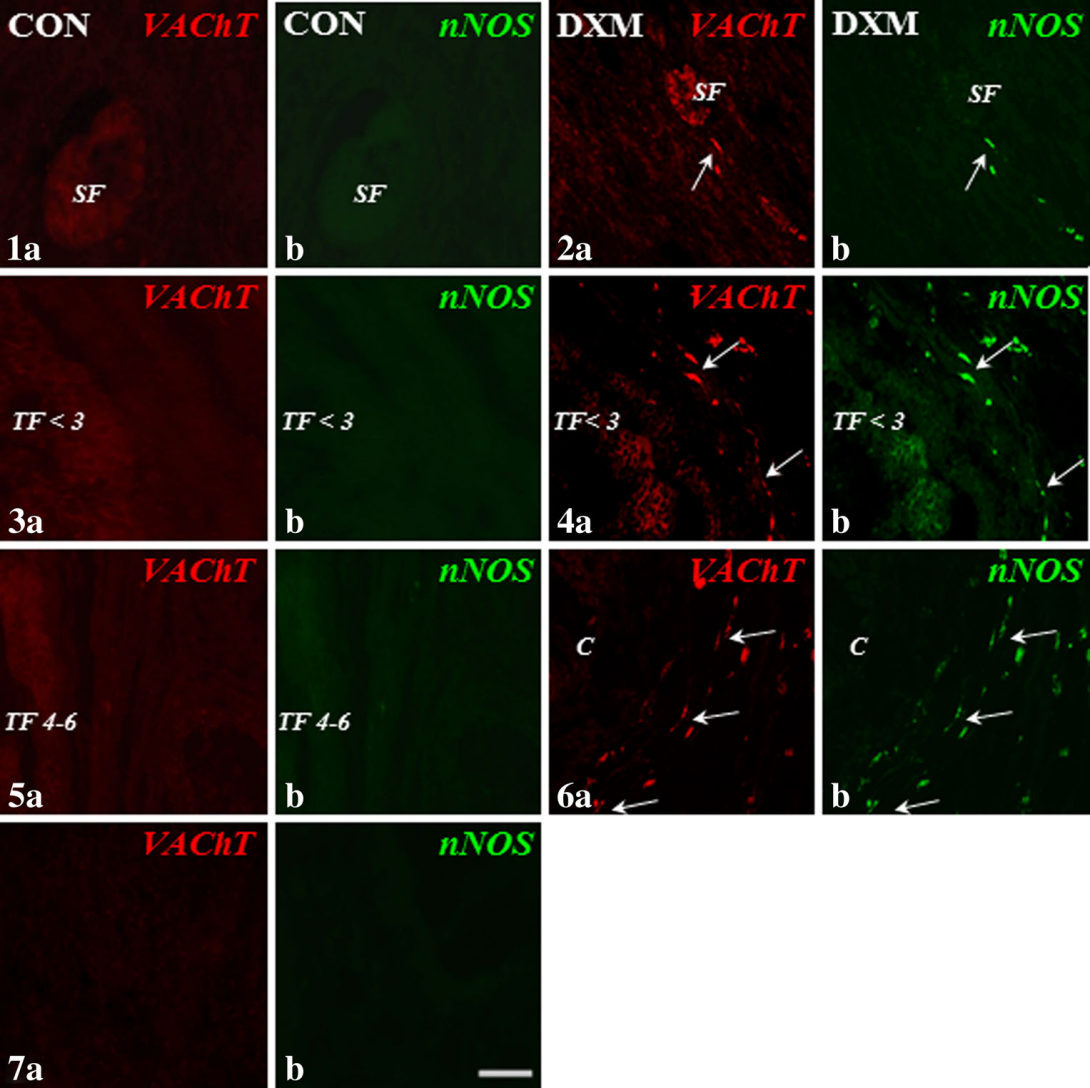
Immunohistochemical localization of VAChT- and nNOS-IR nerve fibres in the cortical part of the ovary of the control (CON) and DXM-treated (DXM) gilts. Lack of VAChT and/or nNOS-IR nerve fibres around the secondary (**1a** and **b**, respectively) and tertiary follicles (up to 3 mm; **3a** and **b**, respectively; 4–6 mm in diameter; **5a** and **b**, respectively) in the CON group. Nerve fibres containing VAChT and/or nNOS near the secondary (**2a** and **b**, respectively) and tertiary up to 3 mm (**4a** and **b**, respectively) follicles and cyst (**6a** and **b**, respectively) in the DXM group. The co-localization of VAChT and nNOS in the same nerve fibres supplying secondary (**2a** and **b**) and tertiary up to 3 mm (**4a** and **b**) follicles and cyst (**6a** and **b**) in the cystic-changed ovaries. Negative control for VAChT (**7a**) and nNOS (**7b**) in the ovary of the CON gilt. *Arrows*, nerve fibres; *SF* secondary follicle, *TF* <*3* tertiary follicle up to 3 mm in diameter, *TF 4*–*6* tertiary follicle 4–6 mm in diameter, *C* cyst. *Scale bar* 25 μm

Compared to the tertiary follicles measuring 4–6 mm in the CON group (Figs. 5a—VAChT, 5b—nNOS and 27—SOM), the presence of nerve fibres storing VAChT (Fig. 6a), nNOS (Fig. 6b) and SOM (Fig. 28) around cysts was revealed. In the ovaries of DXM-treated gilts, in contrast to the CON group (Figs. 1a—VAChT, 1b—nNOS, 3a—VAChT, 3b—nNOS and 25—SOM), VAChT- and nNOS-IR fibres were found near the secondary (Fig. 2a, b; respectively) and tertiary follicles measuring up to 3 mm (Fig. 4a, b; respectively), while SOM-IR fibres were visible within the ground plexus (Fig. 26) and around the tertiary follicles measuring up to 3 mm. Furthermore, the populations of the VIP-IR nerve fibres created thick fascicles and more were found within the ground plexus (P < 0.001; Fig. 13) and in the vicinity of the cysts (*P* < 0.05; Fig. 19) after DXM administration than in the CON group (Figs. 12 and 18, respectively). Neither VAChT- and nNOS-IR nerves supplying arteries nor VIP-IR terminals supplying primordial (Fig. 15) and secondary follicles (Fig. 17) and veins (Fig. 21) were found in the cystic ovaries, when compared to the CON group (Figs. 14, 16 and 20, respectively). Application of DXM did not significantly change the population of VAChT- or nNOS-IR nerve fibres within the ground plexus as well as the number of VAChT-IR fibres around the veins. The density of VIP-containing fibres near the tertiary follicles (measuring to 3 mm in diameter) and arteries as well as VAChT, nNOS, VIP and SOM supplying the interstitial gland was similar in the CON and DXM groups. CLs, present only in the control ovaries, were sporadically innervated by single VAChT-IR nerve fibres. No immunostaining for VAChT (Fig. 7a), nNOS (Fig. 7b), VIP (Fig. 22) and SOM (Fig. 29) was detected in the control ovaries when the primary antibodies were substituted with normal IgGs (in relation to VAChT—rabbit, nNOS and VIP—mouse and SOM—rat).

#### Medulla

**Figs. 8–11:**
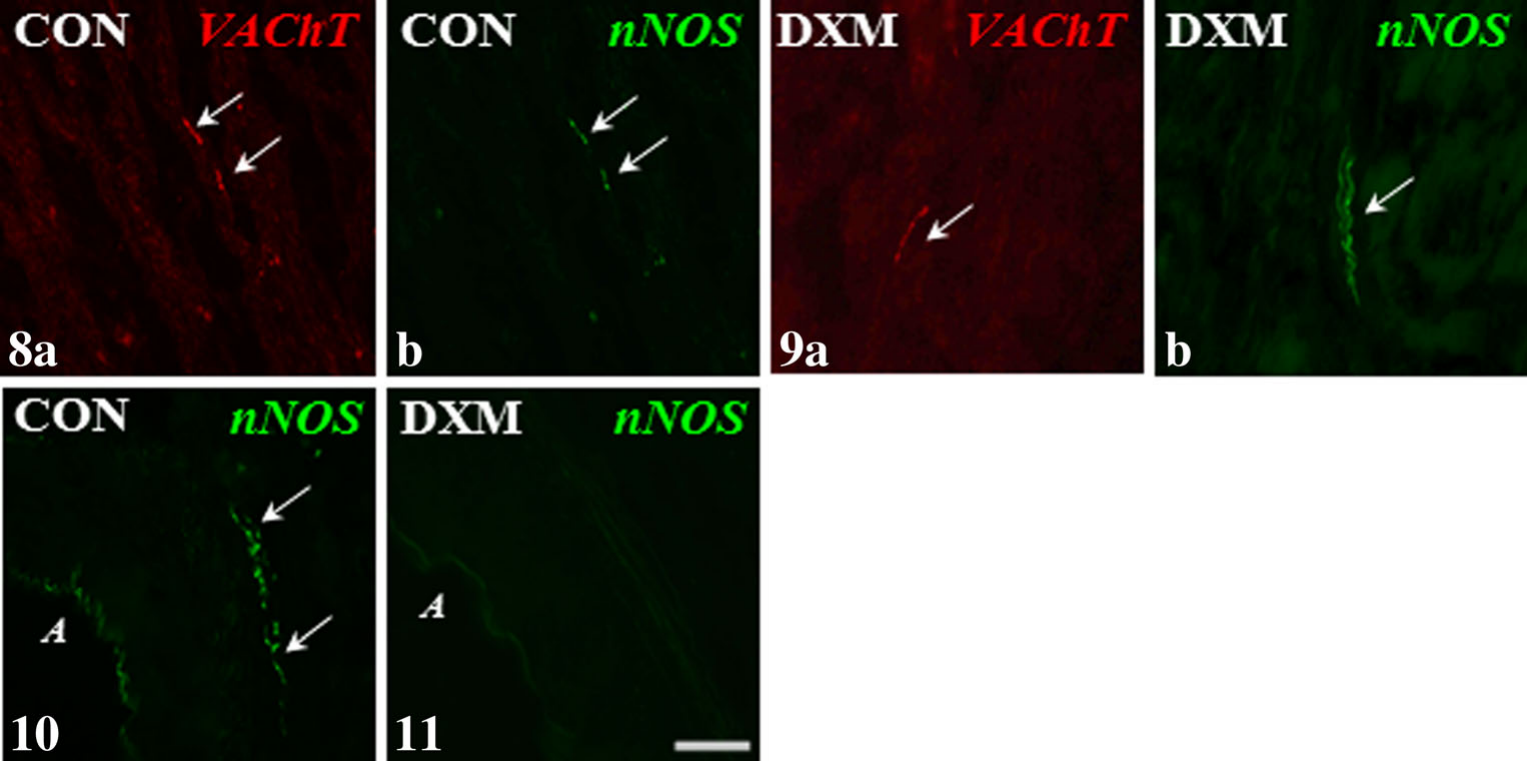
Immunohistochemical localization of VAChT- and nNOS-IR nerve fibres in the medullar part of the ovary of control (CON) and DXM-treated (DXM) gilts. A decrease in the number of VAChT-IR nerve fibres in the area of the ground plexus in the DXM group (**9a**) compared to the CON group (**8a**). Nerve fibres containing nNOS observed in the area of this structure in the CON group (**8b**) and the same number of these nerve fibres forming bundles (**9b**) found after DXM injections. The presence nNOS-IR nerve fibres in the vicinity of an artery in the control gilt (**10**) and their lack in the ovary of DXM-treated animal (**11**). Co-localization of VAChT and nNOS in the same nerve fibres in the area of the ground plexus (**8a** and **b**) in the CON group. *Arrows*, nerve terminal; *A* artery. *Scale bar* 25 μm

In the DXM group, the numbers of nerve terminals expressing VIP (Fig. 24) and SOM (Fig. 31) in the area of the ground plexus were higher (*P* < 0.001), whereas the number of VAChT-IR fibres (Fig. 9a) was lower (*P* < 0.01) than those found in the CON group (Figs. 23—VIP, 30—SOM and 8a—VAChT, respectively). Moreover, following DXM administration, nNOS-IR terminals were not present around arteries (Fig. 11), while these were present in the CON group (Fig. 10). The populations of nNOS-IR nerve fibres within the ground plexus in the CON and DXM groups were similar. Furthermore, these fibres formed fascicles in the cystic ovaries (Fig. 9b), in contrast to the control gonad (Fig. 8b). We did not observe any differences in the number of VIP-IR around the blood vessel, and VAChT- and SOM-IR fibres were not found. Moreover, veins were not supplied by nNOS-IR nerve fibres in all the gilts studied.

### Co-localization Patterns of VAChT and/or nNOS, VIP and SOM Within Nerve Fibres of the Control and Cystic Ovaries

Data concerning the co-localization patterns of VAChT and/or nNOS, VIP and SOM within nerve fibres are summarized in Table 2.

**Table 2:** The co-localization of VAChT and/or nNOS, VAChT and/or VIP and VAChT and/or SOM in the porcine ovaries of the control (CON) and DXM-treated (DXM) groups

	Ovarian tissue	Group	VAChT and/or nNOS	VAChT and/or VIP	VAChT and/or SOM
Cortex	GP	CON	col.	–	–
		DXM		–	–
	PF	CON	–	–	–
		DXM	–		–
	PRF	CON	–	–	–
		DXM	–	–	–
	SF	CON	–	–	–
		DXM	col.	–	–
	TF to 3 mm	CON	–	–	–
		DXM	col.	–	–
	TF 4–6 mm	CON	–	–	–
		DXM	l.s.	l.s.	l.s.
	C	CON	l.s.	l.s.	l.s.
		DXM	col.	–	–
	CL	CON	–	–	–
		DXM	l.s.	l.s.	l.s.
	A	CON	col.	–	–
		DXM	–	–	–
	V	CON	–	–	–
		DXM	col.	–	–
	IG	CON	–	–	–
		DXM	–	–	–
Medulla	GP	CON	col.	–	–
		DXM	–	–	–
	A	CON	–	–	–
		DXM	–	–	–
	V	CON	–	–	–
		DXM	–	–	–

*GP* ground plexus, *PF* primordial follicles, *PRF* primary follicles, *SF* secondary follicles, *TF* tertiary follicles, *C* cysts, *CL* corpora lutea, *A* arteries, *V* veins, *IG* interstitial gland, *l.s*. lack of structures, *col*. co-localization in the same nerve fibres of both substances studied, – lack of co-localization in the same nerve fibres of both substances studied

#### Cortex

In both the CON and DXM groups, a part of VAChT-IR nerve fibres within the ground plexus was simultaneously nNOS positive. Some nerve fibres around cysts containing VAChT and nNOS (Fig. 6a, b), while the co-localization of these substances was not observed in fibres near the tertiary follicles measuring 4–6 mm in the CON group (Fig. 5a, b). A similar result was found for the VAChT- and nNOS-IR nerve fibres projecting to the secondary (Fig. 1a, b—CON group; Fig. 2a, b—DXM group) and tertiary follicles measuring up to 3 mm (Fig. 3a, b—CON group; Fig. 4a, b—DXM group) as well as the veins. In turn, a part of the fibres near the arteries in the CON group was VAChT- and nNOS-IR, which was not found in the cystic ovaries. In both the CON and DXM groups, the co-localizations of VAChT/VIP and VAChT/SOM were not observed in the fibres innervating all the studied ovarian structures.

#### Medulla

In the CON group, a part of the nerve fibres in the area of the ground plexus contained simultaneously VAChT and nNOS (Fig. 8a, b), while in the DXM group, these substances did not occur together (Fig. 9a, b). In the control and cystic ovaries, the co-localization of VAChT and nNOS in the fibres around blood vessels was not revealed. A similar situation was observed for VAChT and VIP as well as VAChT and SOM in the fibres within the ground plexus and projecting to the blood vessels in both studied groups (Figs. 1–7, 8–11, 12–22, 23-24, 25–29 and 30–31).

**Figs. 12–22:**
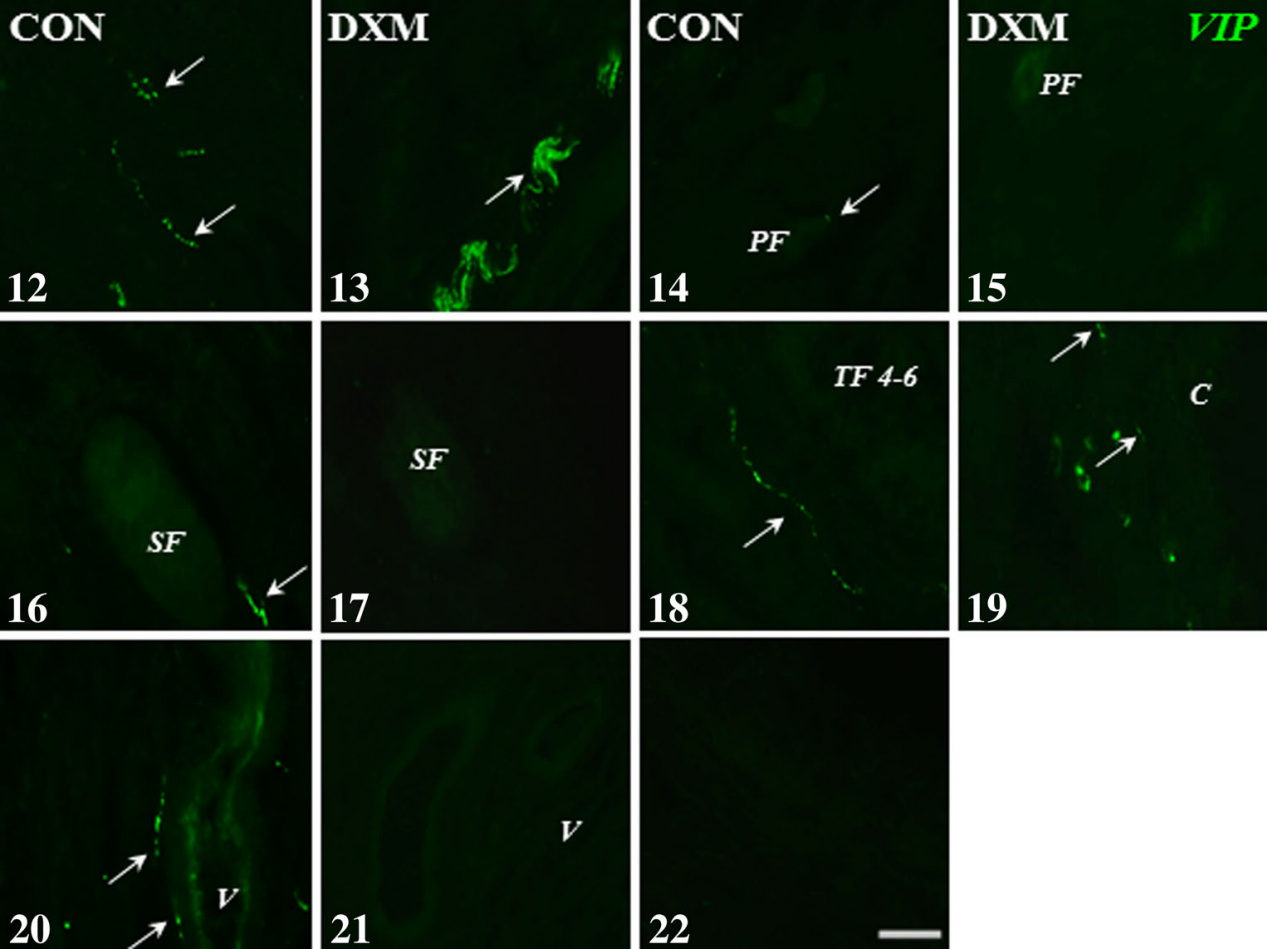
Immunohistochemical localization of VIP-IR nerve fibres in the cortical part of the ovary of the control (CON) and DXM-treated (DXM) gilts. An increase in the number of VIP-positive nerve fibres, formed bunches, in the area of the ground plexus (**13**) as well as near the cyst (**19**) in the ovary of the DXM-treated animal compared to the CON group (**12** and **18**, respectively). A lack of VIP-IR nerve fibres around the primordial (**15**) and secondary (**17**) follicles as well as the vein (**21**) after DXM administration and their presence in the CON group (**14**, **16** and **20**, respectively). Negative control for VIP in the ovary of the CON gilt (**22**). *Arrows*, nerve fibres; *PF* primordial follicle, *SF* secondary follicle, *TF 4*–*6* tertiary follicle 4–6 mm in diameter, *C* cyst, *V* vein. *Scale bar* 25 μm

**Figs. 23–24:**
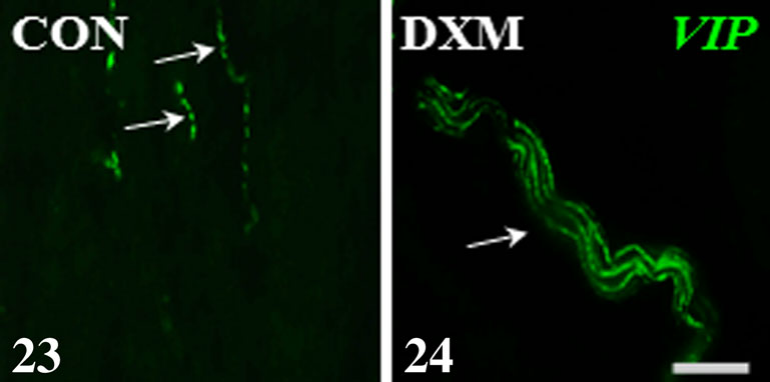
Immunohistochemical localization of VIP-IR nerve fibres in the medullar part of the ovary of the control (CON) and DXM-treated (DXM) gilts. An increase in the number of VIP-IR nerve fibres, often forming bundles, in the area of the ground plexus in the cystic-changed ovary (**24**) compared to the control gonad (**23**). *Arrows*, nerve fibres. *Scale bar* 25 μm

**Figs. 25–29:**
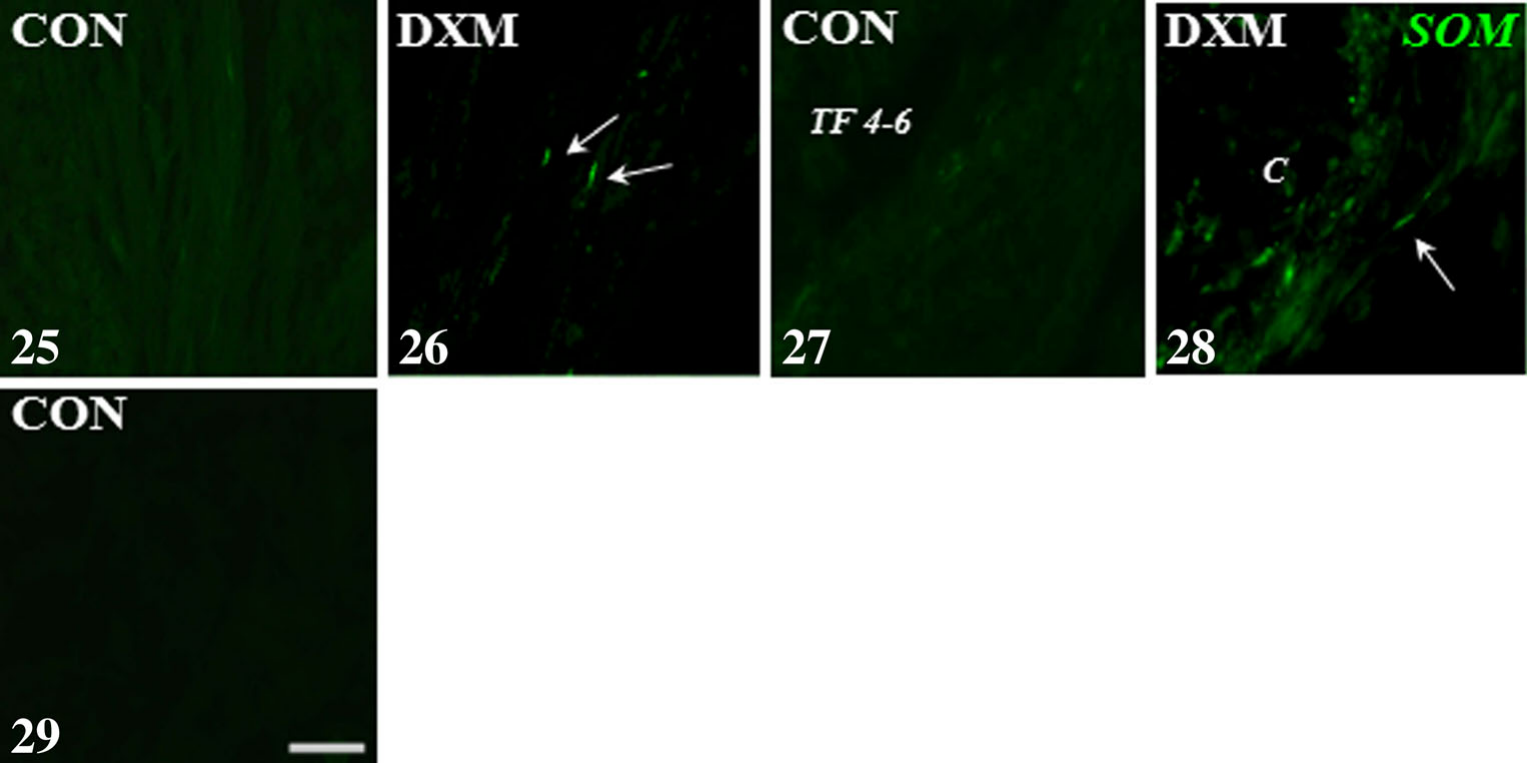
Immunohistochemical localization of SOM-IR nerve fibres in the cortical part of the control (CON) and DXM-treated (DXM) gilts. SOM-IR nerve fibres were not observed within the ground plexus (**25**) as well as around the tertiary follicles (4–6 mm in diameter; **27**) in the control gilts, while they were present in the ovaries of DXM-treated gilts (**26** and **28**, respectively). Negative control for SOM (**29**) in the ovary of the CON gilt. *Arrows*, nerve fibres; *TF 4*–*6* tertiary follicle 4–6 mm in diameter, *C* cyst. *Scale bar* 25 μm

**Figs. 30–31:**
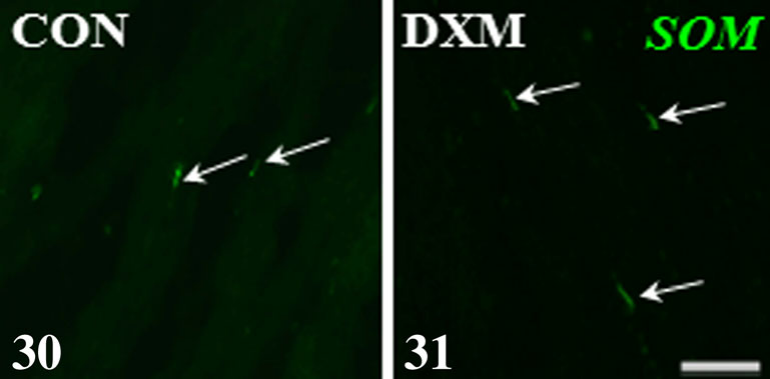
Immunohistochemical localization of SOM-IR nerve fibres in the medullar part of the control (CON) and DXM-treated (DXM) gilts. An increase in the number of the SOM-IR nerve fibres in the area of the ground plexus in the DXM group (**31**) compared to the CON group (**30**). *Arrows*, nerve fibres. *Scale bar* 25 μm

## Discussion

The present experiment revealed that the distribution and density of nerve fibres containing VAChT, nNOS, VIP and/or SOM have changed in the gilt cystic ovaries, induced by DXM treatment on days 7–21 of the estrous cycle. After DXM administration, we have observed the presence of VAChT-, nNOS- and SOM-IR nerve fibres around the cysts and tertiary follicles measuring up to 3 mm in diameter. nNOS- and/or VAChT-IR fibres were found also near the secondary follicles and veins, while SOM-IR fibres were within the ground plexus. In turn, in the cortical part of cystic ovaries, the lack of VAChT- and nNOS-IR nerve fibres in the vicinity of the arteries and VIP-IR endings enclosing the veins was revealed. These results are partly different from those observed previously in the DXM-induced cystic ovaries from the follicular phase of the estrous cycle (Kozłowska et al. 2008). Thus, in the present study, VAChT-IR nerve terminals around cortical arteries and VIP-IR nerve fibres in the vicinity of cortical veins were observed in both groups (the CON and DXM), while a lack of VAChT- and nNOS-IR nerve fibres supplying veins and SOM-IR near cysts was reported after DXM treatment (Kozłowska et al. 2008). Additionally, in the porcine cystic gonads, examined in the present study, an increase in the number of VIP-IR nerve terminals, often forming bundles, was observed around cysts and within the ground plexus in the whole ovary. Furthermore, we have observed an increase in the density of SOM-IR fibre population within the medullar part of the ground plexus, while simultaneously the number of VAChT-IR nerve fibres decreased in the latter structure. The increased population of VIP-IR nerve fibres within the medullar part of cystic ovaries was earlier observed following injection of DXM from the follicular phase of the estrous cycle (Kozłowska et al. 2008), and it has also been shown that the concentration of VIP rose markedly in the estradiol valerate (EV)-induced polycystic ovaries (Parra et al. 2007). However, in the DXM-treated gilts from the follicular phase, in contrast to the current report, the density of VIP-IR nerve fibres near cysts and within the cortical part of the ground plexus as well as VAChT-IR in the area of the medullar part of the ground plexus was similar to those revealed in the CON group (Kozłowska et al. 2008). Furthermore, VIP-IR nerve fibres were not observed around the primordial and secondary follicles and cortical veins in the present experiment, in contrast to the situation found after induction of cystic changes from the follicular phase of the cycle (Kozłowska et al. 2008). We demonstrated also (in the present study) that the nerve fibres containing VAChT were negative for VIP and SOM, while the fibres exhibiting VAChT/VIP and VAChT/SOM were observed in the ovaries of the DXM-treated gilts from the follicular phase of the cycle (Kozłowska et al. 2008).

Explanation of the results obtained in the present study is difficult because in the literature there is only one report about the cholinergic innervation of cystic ovaries. We assume that the appearance and/or increase in the density of nerve fibres supplying some structures in the cystic gonads may be related to a local ovarian mechanism that results in neuronal plasticity. Thus, it may converge with the higher production of nerve growth factor (NGF), which plays a crucial role in growth, maintenance and survival of nerve cells and in the regulation of axon and dendrite growth (Huang and Reichardt 2001). It has earlier been revealed that the level of NGF and its receptor (p75) was enhanced in rat EV-induced polycystic ovaries (Lara et al. 2000) and porcine cystic ovaries evoked by DXM (Jana et al., unpublished data). On the other hand, the changed chemical coding of neurons may be a result of the direct effects of steroid hormones on these cells as well. It is recognized that estrogen receptors are expressed in the ovarian neurons of sympathetic and parasympathetic as well as sensory ganglia in adult gilts and that the long-term 17β-estradiol treatment reduced the population of these neurons (Koszykowska et al. 2011a, b; Jana et al. 2012, 2013a). A similar situation has been revealed for neurons in the caudal mesenteric ganglion innervating the ovary in adult gilts following testosterone administration (Jana et al. 2013b). It is worth noting that in the gilts, from which the cystic ovaries were collected for the present study, the peripheral blood levels of sex steroids were markedly changed (Jana, unpublished data). The changes in the distribution and/or number of cholinergic nerve fibres in the cystic porcine ovaries (present study) can also be the consequence of the DXM effect per se. In rodents, DXM enhanced the cholinergic nerve terminal development in the brain (Zahalka et al. 1993), the expression of nNOS mRNA in neuroblastoma cells (Schwarz et al. 1998) and the growth of VIP mRNA/protein in the Langerhans islets (Jamal et al. 1991).

Considering the changes in the cholinergic innervation pattern of the cystic ovaries revealed in the present study, we suppose that they may have importance in the function of pathologically-changed gonads. It has been reported that under physiological conditions, ACh acting by muscarinic receptor modulates the ovarian blood flow (Dynarowicz and Dzięgielewski 1987), the follicular development and steroidogenesis (Delgado et al. 2010; Daneri et al. 2013) as well as takes part in the ovulation (Walles et al. 1976). In addition, NO (Masuda et al. 2001; Barszczewska and Jaroszewski 2004), VIP (Miyamoto et al. 1993; Hulshof et al. 1994) and SOM (Panconesi et al. 1987; Andreani et al. 1995) may participate in the ovarian blood flow and/or steroidogenesis.

Our earlier (Kozłowska et al. 2008) and present studies show partial differences in the cholinergic innervation patterns of the ovaries with cystic changes induced by DXM treatment from the follicular or middle luteal phase of the estrous cycle, respectively. This situation may result from the other phase of the estrous cycle (different stage of follicular development and hormonal status) in which the administration of DXM was initiated. It has been reported that application of adrenocorticotropic hormone or glucocorticoids in pigs before or after the time of ovulatory follicle selection resulted in differential macroscopic and steroid hormone changes of cystic ovaries (Gee et al. 1991). Moreover, the activity of acetylcholine esterase in the porcine ovaries changed during the estrous cycle (Łakomy et al. 1984) as well as the expression of endothelial NOS (Chatterjee et al. 1996) and the concentration of VIP (Bruno et al. 2011) in rat ovaries. Taking these data into consideration, we can suggest that the changes observed in cholinergic innervation revealed between the cystic ovaries, induced by DXM from the middle luteal (present study) and follicular (Kozłowska et al. 2008) phase of the estrous cycle, may depend on the phase of the cycle, in which the activation of the hypothalamic-pituitary-adrenal axis and cholinergic neurons occurred. However, this assumption must be further elucidated in detail.

In summary, the present study shows changes in the distribution and number of nerve fibres containing VAChT and/or nNOS, VIP and SOM in the gilt ovaries with DXM-induced status polycysticus according to the kind of chemical coding of fibre and/or ovarian structure. The data obtained suggest an important role of ACh, NO, VIP and SOM in the course of this pathological state. Our findings confirm that the morphological changes of cystic ovaries are partly dependent on phase of the estrous cycle in which the induction of the cysts was started.
